# Differentiation of Chronic Lymphocytic Leukemia B Cells into Immunoglobulin Secreting Cells Decreases LEF-1 Expression

**DOI:** 10.1371/journal.pone.0026056

**Published:** 2011-10-06

**Authors:** Albert Gutierrez, Bonnie K. Arendt, Renee C. Tschumper, Neil E. Kay, Clive S. Zent, Diane F. Jelinek

**Affiliations:** 1 Department of Immunology, Mayo Graduate School, College of Medicine, Mayo Clinic, Rochester, Minnesota, United States of America; 2 Department of Internal Medicine, Mayo Graduate School, College of Medicine, Rochester, Mayo Clinic, Minnesota, United States of America; Roswell Park Cancer Institute, United States of America

## Abstract

Lymphocyte enhancer binding factor 1 (LEF-1) plays a crucial role in B lineage development and is only expressed in B cell precursors as B cell differentiation into mature B and plasma cells silences its expression. Chronic lymphocytic leukemia (CLL) cells aberrantly express LEF-1 and its expression is required for cellular survival. We hypothesized that modification of the differentiation status of CLL cells would result in loss of LEF-1 expression and eliminate the survival advantage provided by its aberrant expression. In this study, we first established a methodology that induces CLL cells to differentiate into immunoglobulin (Ig) secreting cells (ISC) using the TLR9 agonist, CpG, together with cytokines (CpG/c). CpG/c stimulation resulted in dramatic CLL cell phenotypic and morphologic changes, expression of cytoplasmic Ig, and secretion of light chain restricted Ig. CpG/c stimulation also resulted in decreased CLL cell LEF-1 expression and increased Blimp-1 expression, which is crucial for plasma cell differentiation. Further, Wnt pathway activation and cellular survival were impaired in differentiated CLL cells compared to undifferentiated CLL cells. These data support the notion that CLL can differentiate into ISC and that this triggers decreased leukemic cell survival secondary to the down regulation of LEF-1 and decreased Wnt pathway activation.

## Introduction

Chronic lymphocytic leukemia (CLL) is a clonal expansion of neoplastic mature B lymphocytes and is the most prevalent lymphoid malignancy in the United States. CLL is effectively treated with chemo-immunotherapy although CLL remains an incurable malignancy with conventional management. To better understand this disease, state of the art genomic methodologies have been employed[1], [2], [3], [4] to identify genes involved in disease pathogenesis. Despite new insights emerging from genome-wide studies, there is still a need to further explore novel therapeutic alternatives to improve the treatment of this malignancy.

We and others have previously shown CLL cells aberrantly express the transcription factor lymphoid enhancer binding factor-1 (LEF-1) and rely on this factor for their survival.[5], [6] LEF-1 is a target gene and central mediator of the wingless-type MMTV integration site (Wnt) signaling pathway, which plays an active role in the biology of CLL.[7] While LEF-1 is critical during normal B cell development in the bone marrow,[8] LEF-1 expression is restricted to developing B cells, with expression turned off at later stages of B lineage development, i.e., mature B cells and plasma cells.[9] In a mechanism(s) yet to be identified, CLL cells have reacquired expression of this developmentally important survival factor. We hypothesized that differentiation of leukemic B cells into immunoglobulin (Ig) secreting cells (ISC) would result in loss of LEF-1 expression and decreased leukemic cell survival.

Agents that induce terminal differentiation of leukemic cells have been utilized therapeutically with the greatest successes realized using all-*trans* retinoic acid for acute promyelocytic leukemia and interferon-α for hairy cell leukemia.[10], [11] This strategy relies on the fact that leukemic cells are blocked at a particular stage in their development and are not truly terminally differentiated. Several agents are able to induce leukemic cells to overcome their block of differentiation resulting in inhibition of proliferation and an increase in apoptotic cell death. This alternative differentiation strategy has been combined with traditional cytotoxic therapies to increase the efficacy of treatment.

Of relevance to this study, there has been an interest in utilizing toll like receptor (TLR) agonists in the treatment of hematologic malignancies.[12], [13], [14] TLRs are a family of pattern recognition receptors involved in the detection of pathogen associated molecular patterns and other “danger” signals. This large class of receptors plays a crucial role in both innate and adaptive immunity. The rationale for their use in hematologic malignancies has centered on the induction of and sensitization to the anti-leukemic immune response, as well as potential direct anti-leukemic properties of these agents.[14]

Previous studies have shown that CLL cells respond to the TLR9 agonist, CpG, with proliferation, upregulation of costimulatory molecules, and induction of apoptosis.[15], [16], [17] However, to our knowledge there has been no study to date that has evaluated the ability of these agents to induce CLL B cell differentiation into ISC. Previous studies suggest that CLL cells can secrete antibody in response to certain stimuli such as pokeweed mitogen, or other stimuli with cytokines.[18], [19], [20], [21], [22] TLR agonists, along with cytokines IL-2 and IL-15, are well characterized in their ability to induce terminal differentiation of normal mature B cells.[23] We hypothesized that CpG along with cytokines IL-2 and IL-15 (CpG/c) could be used as a tool to induce CLL cell differentiation and alter prosurvival signaling by LEF-1 and the Wnt pathway. In the present study, we addressed two critical questions; (1) do CLL cells lose LEF-1 expression upon differentiation into ISC; and (2) do CLL cells that differentiate into ISC lose LEF-1 prosurvival signaling and exhibit decreased survival?

## Methods

### Ethics statement and patient cohort

Mayo Clinic Institutional Review Board (IRB) approval was obtained for use of human blood from healthy donors. The IRB reviewed our request to use blood from healthy donors and specifically waived the need for informed consent for our protocol due to the ruling that this is waste material generated during blood donation. In further support of the IRB waiver of informed consent, these specific patient samples arrive de-identified in the laboratory. Regarding CLL patients, Mayo Clinic IRB approval was obtained and blood was only used from patients providing written informed consent in accord with Helsinki protocol. Patient clinical and relevant prognostic factors are summarized in Table 1.

**Table 1:** Patient cohort characteristics.

Patient	Age/Sex	Stage	CD38	ZAP-70	IGHV gene/ % Mutation	FISH	Prior Tx*
1	60/M	I	neg	neg	3–53/12%	13q-	no
2	75/M	II	neg	POS	3–33/9.8%	13q-	no
3	68/F	0	neg	neg	3–7/7.3%	Normal	no
4	66/M	0	neg	neg	4–59/11.7%	13q-	no
5	69/M	IV	neg	POS	1–3/0.0%	Normal	PCR
6	45/M	I	POS	neg	7–4/0.0%	13q-	no
7	56/M	II	neg	POS	1–69/0.0%	11q-	FCR
8	56/M	II	neg	POS	3–23/2.6%	13q-	no
9	56/F	I	neg	neg	1–18/0.0%	11q-, 13q-	AR
10	65/M	0	neg	neg	4–4/12.5%	13q-	no
11	58/M	0	neg	neg	4–39/12.8%	Normal	no
12	64/F	I	neg	POS	3–9/0.0%	Normal	no
13	74/F	I	neg	neg	4–34/0.0%	Trisomy 12	no
14	86/F	0	neg	neg	3–7/6.3%	Normal	no
15	64/F	0	neg	neg	4–34/6%	13q-	no
16	69/M	I	neg	neg	1–08/4.5%	13q-×2	no
17	78/F	0	neg	neg	1–08/4.3%	13q-×2	no
18	58/M	II	neg	neg	3–11/8.1%	Normal	no
19	72/M	II	neg	POS	3–49/0.0%	17p-, 13q-	no
20	49/M	I	neg	POS	1–69/0.0%	Normal	no
21	60/M	0	neg	neg	3–7/4.9%	13q-	no
22	70/M	0	POS	neg	3–30/0.0%	Trisomy 12	no
23	81/F	II	neg	POS	3–07/4.5%	17p-, 13q-, t(14;18)	AR, PAR
24	85/F	0	neg	neg	1–18/4.7%	t(14;18)	no
25	73/M	IV	POS	POS	5–51/0.0%	Trisomy 12, t(14;19), +14q32 (IGH+)	PR, PCR

*Treatments abbreviated as follows; P is pentostatin, C is cyclophosphamide, R is rituximab, F is fludarabine, and A is alemtuzumab.

### Cell isolation and culture

Peripheral blood mononuclear cells (PBMC) from CLL patients or healthy donors were separated from heparinized venous blood by Ficoll density gradient centrifugation. The PBMC used in this study were generally greater than 90% CLL B cells as determined by CD19/CD5 positivity. Leukemic cells were cultured in serum-free adoptive immunotherapy media-V (AIM-V) medium (Gibco-Invitrogen). For CLL B cell activation experiments, cells were cultured for 5 days in AIM-V media or AIM-V media supplemented with 2.5 µg/ml CpG ODN 2006 (provided by core Mayo Clinic facility) with or without 94 IU/ml human IL-2 (Peprotech), 10 ng/ml human IL-15 (Peprotech), and 13 µM beta-mercaptoethanol. Further CLL B cell activation experiments utilized 12-O-tetradecanoylphorbol-13-acetate (TPA) (Sigma) at 10 ng/ml or interferon-α 2b (Schering Corp.) at 1000 IU/ml. Human CD4+T cells were isolated from healthy donor PBMC by immunomagnetic selection using T cell enrichment kits (StemCell Technologies) and incubated with CLL B cells at a ratio of 1∶2 in plates pre-coated with PBS containing 10 µg/ml of anti-CD3 (R&D) and anti-CD28 (BD) stimulating antibodies. All cells were maintained in appropriate media at 37°C in an atmosphere containing 95% air and 5% CO_2_.

### Quantitative polymerase chain reaction (qPCR)

Sense and antisense primers to *LEF1* (forward- 5′-GAC GAG ATG ATC CCC TTC AA-3′/reverse- 5′-AGG GCT CCT GAG AGG TTT GT-3′) and 18 s rRNA (forward- 5′-AAA CGG CTA CCA CAT CCA AG-3′/reverse- 5′-CCT CCA ATG GAT CCT CGT TA-3′) were designed using Primer3. *PRDM1* primers were purchased from SABiosciences. Total RNA isolated by the Trizol method (Invitrogen) was reverse transcribed to cDNA using the 1 st Strand cDNA Synthesis kit (GE Healthcare). One µl of cDNA was amplified using the SYBR Green/Rox PCR reagent (SABiosciences) in a total volume of 20 µl, which included 5 pM of each primer and 1 x SYBR Green Mastermix. Amplification was carried out using a 7900 HT Applied Biosystems fast real-time PCR system as follows: denaturation at 95°C for 15 min; 40 cycles of 15 s at 95°C; 60 s at 60°C and a melting curve cycle. Melting temperature and quantitative analysis was performed using SDS RQ software. Relative fold change was normalized against 18 s rRNA after a 1∶2000 cDNA dilution and calculated using the 2^−ΔΔCT^ method.

### Western blot analysis

Cells were lysed for 30 min on ice in lysis buffer containing 10 mM Tris (pH 7.4), 150 mM NaCl, 1% Triton X-100, 0.5% deoxycholate, 0.1% SDS, 5 mM EDTA, complete mini protease inhibitor cocktail (Roche), and phosSTOP phosphatase inhibitor cocktail (Roche). An equal volume of Laemmli sample buffer (Bio-Rad) was added before boiling for 5 min. Lysates were subjected to electrophoresis through a 10% Tris-HCl polyacrylamide gel using Tris-Glycine-SDS buffer. Proteins were transferred to PVDF membranes (Millipore) and blocked with 25 mM Tris-HCl (pH 7.2), 150 mM NaCl, and 0.1% Tween 20 (TBST) plus 5.0% Blotto (ISC BioExpress). The blot was probed with anti-LEF-1 antibody (Cell Signaling Technology) at a 1∶1000 dilution, or anti-β-actin antibody (Novus) at a 1∶5000 dilution. After three washes with TBST, donkey anti-rabbit or sheep anti-mouse HRP secondary (GE Healthcare) was used at a 1∶2000 dilution. After washing, SuperSignal (Pierce) Chemiluminescent substrate was then used to detect proteins.

### Flow cytometry

Cells were washed in FACS buffer (PBS+1% FCS) and incubated with conjugated primary antibody for 10 minutes at room temperature. Antibodies included anti-CD19 APC and anti-CD5 PE (BD Biosciences). For intracellular staining, cells were fixed in 4% formaldehyde/PBS at 37°C for 10 minutes and permeabilized by resuspending in 90% methanol and incubation on ice for 30 minutes. Cells were incubated with primary anti-LEF1 antibody (Cell Signaling) or isotype control for 1 hr, washed, and incubated with secondary Alexa-488 goat anti-rabbit IgG (Invitrogen) for 30 minutes. Cells were then analyzed on a BD FACSCalibur and data analysis was performed using FlowJo software (TreeStar).

### ELISA

Following 5 days of culture under the conditions described above, cell free culture supernatants were collected and analyzed for levels of secreted Ig using IgM, IgG, kappa, and lambda ELISA. Briefly, 96-well microtiter plates (Nalge Nunc International) were independently coated with anti-IgG, anti-IgM (BioSource International), anti-kappa, or anti-lambda (Bethyl Laboratories) antibodies. Plates were then blocked with 1x casein (BioFX Laboratories). After several washes, culture supernatants were added to coated plates and incubated for 2 h. Igs were detected colorimetrically using anti-IgG, anti-IgM (BioSource International), anti-kappa, or anti-lambda (Bethyl Laboratories) HRP-labeled antibodies and a Molecular Devices microplate reader. Standard curves were generated to quantitate ELISA results using known amounts of purified human IgG and IgM (Jackson ImmunoResearch Laboratories), as well as purified kappa, and lambda light chain proteins (Bethyl Laboratories). o-Phenylenediamine dihydrochloride ELISA substrate for HRP along with stable peroxide substrate buffer were purchased from Pierce.

### TCF/LEF dual luciferase assay

TCF/LEF reporter plasmids were purchased from SA Biosciences. The positive reporter plasmid has TCF/LEF consensus binding sites that drive expression of firefly luciferase, and the negative reporter lacks TCF/LEF binding sites. These plasmids were introduced along with a constitutively expressed renilla luciferase plasmid into CLL cells as previously described.[5] CLL cells were cultured for 5 days in CpG/c or control media, separated by Ficoll density gradient centrifugation, nucleofected, and then cultured in control media for 48 hours before being harvested. Positive reporter firefly luciferase values were normalized to constitutively expressed renilla luciferase and a negative reporter fold change of 1.

### Cell survival analysis

Cells were either unstimulated or stimulated as described above for 5 days prior to isolation of viable cells using Ficoll density gradient centrifugation. Ficoll-isolated cells were evaluated by trypan blue exclusion to measure cell viability and purity was evaluated by CD19/CD5 FACS analysis before culturing for an additional 48 hours in AIM-V media. Purity ranged from 90–97% CLL cells of Ficoll-isolated cells from 5 day stimulated cultures. On day 7, cell survival was measured by Annexin-FITC (BD Biosciences) and propidium iodide (PI) staining and analyzed by flow cytometry.

### Cytoplasmic Ig (cIg) and morphological studies

CLL cell morphology was analyzed using cytospin preparations of the cells on glass slides made using a Thermo Shandon Cytospin 2 and stained by standard Wright-Giemsa (Fisher). For cIg staining, cells were fixed in 95% ethanol for 5 minutes, air dried, and then permeabilized with 0.1% NP-40 in PBS. Cells were then stained with a FITC-conjugated F(ab’)_2_ polyclonal antibody with specificity for human IgM, IgG, and IgA (Biosource International) at a dilution of 1∶100 in PBS with 5% serum for 45 min at 37° C. The cells were then washed with 0.1% NP-40 in PBS before adding mounting media containing either DAPI or PI and coverslipped (Vector Labs). Cells were viewed by light microscopy (Olympic Provus AX70; Olympus) and images were acquired using an Olympus DP71 microscope digital camera equipped with Olympus DP Manager Software. Using Microsoft PowerPoint, the brightness of all images were uniformly decreased 7%.

### [^3^H]-Thymidine proliferation assay

Proliferation assays were performed as described previously,[24] with the exception that 2×10^5^ CLL cells were stimulated or not as described above in 96 well plates for 72 hours before being pulsed with 1μCi of tritiated thymidine (Amersham). Cells were then cultured for 18 hours, harvested and counted using liquid scintillation spectroscopy.

### Statistical methods

[^3^H]-Thymidine proliferation assays, TCF/LEF reporter assays, and ELISA and survival data were analyzed using a paired t-statistic analysis approach.

## Results

### CpG/c stimulated CLL cells proliferate and develop a plasmacytoid morphology

**Figure 1:**
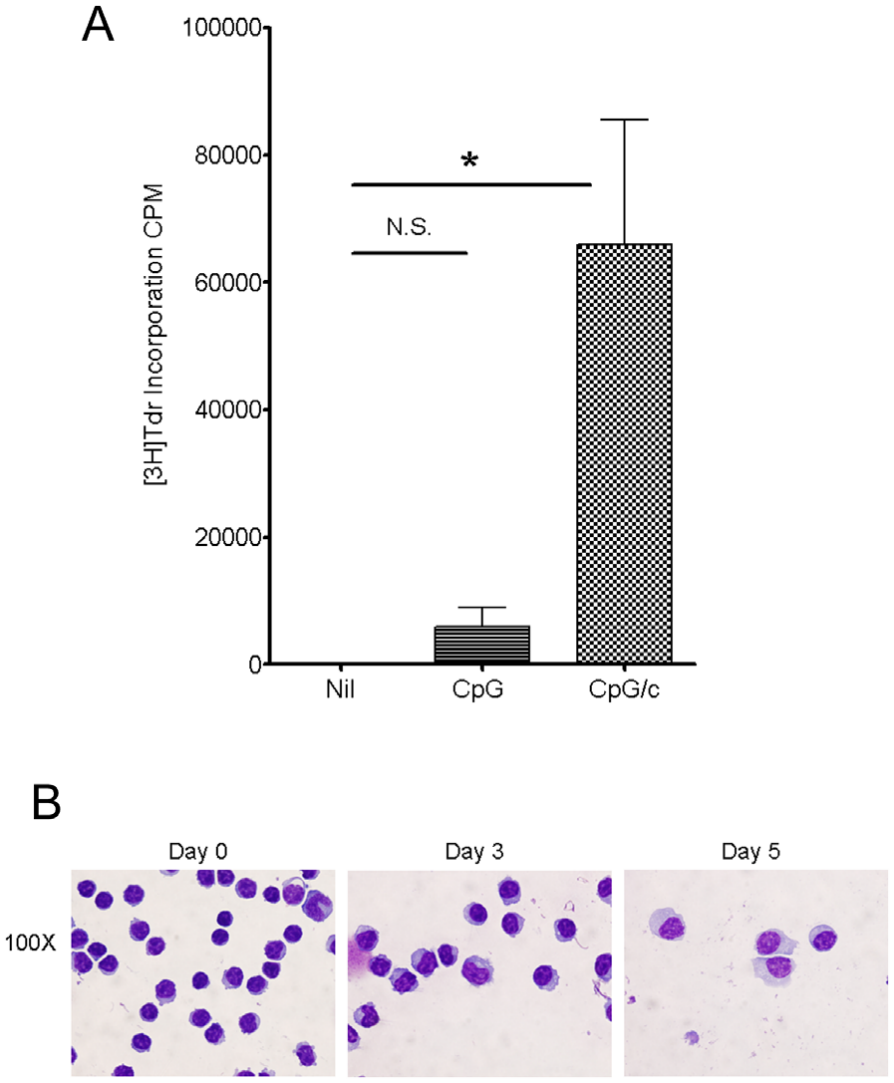
CpG/c stimulation induces proliferation and plasmacytoid morphology in CLL cells. **A.** Detection of proliferation in leukemic B cells from 6 CLL patients by [^3^H]-thymidine incorporation. (*p = .02; error bars represent standard error of the mean) **B.** Morphologic analysis of leukemic B cells on days 0, 3, and 5 after CpG/c stimulation as revealed by Wright Giemsa staining.

Previous studies have used CpG stimulation as a tool to induce primary CLL B cell proliferation[16], [17], [25] and because of this property, CpG stimulation has utility in metaphase analysis of leukemic B cells.[26], [27] Although the ability of CpG stimulation to induce CLL cell differentiation has not been previously reported, it has been shown that CpG/c is effective in inducing the differentiation of normal B cells into ISC.[23], [28] Because the primary goal of our study was to assess the impact of leukemic B cell differentiation on LEF-1 expression, we first wished to determine if CpG/c could similarly induce CLL B cell differentiation. Initially, we analyzed the effects of CpG vs CpG/c on CLL B cell proliferation and observed that CpG/c stimulates a much greater proliferative response than CpG alone (n = 6, p = 0.020, **Fig.1A**). We reasoned that CpG in concert with cytokines might also have the potential to induce CLL cell differentiation into ISC.

To address this possibility, we took advantage of the knowledge that ISC are large cells with a characteristic morphology that includes an abundant cytoplasm and an eccentrically located oval nucleus. Indeed, CLL B cells stimulated for 5 days with CpG/c, took on the characteristic morphology of ISC (**Fig. 1B**). These data suggest that CpG/c stimulation could induce CLL B cell differentiation into ISC.

### CpG/c stimulated CLL B cells increase cIg expression and secrete light chain restricted Ig

**Figure 2:**
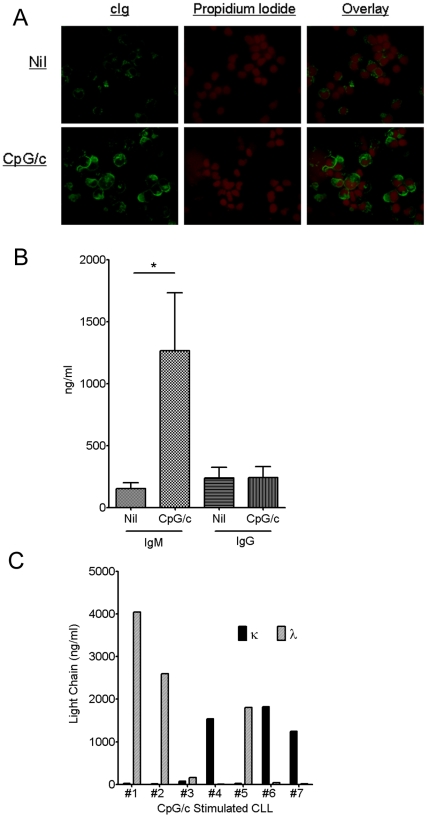
CpG/c stimulated CLL B cells secrete light chain restricted Ig. **A.** Representative propidium iodide and immunofluorescence staining for cytoplasmic Ig in CpG/c stimulated leukemic B cells. **B.** ELISA detection of IgM and IgG levels in cell free culture supernatants from 14 patients in unstimulated or CpG/c treated CLL cultures after 5 days. (*p = .02; error bars represent standard error of the mean) **C.** ELISA detection of kappa and lambda light chain Ig levels in cell free culture supernatants from 7 patients in nil or CpG/c treated CLL cultures after 5 days.

CLL B cell Ig expression is typically restricted to the cell membrane and the majority of patients express membrane IgM and IgD, although at varying levels. Because a hallmark feature of Ig secreting differentiated B cells is the appearance of cIg and switch from membrane expression to secreted forms of Ig, we next evaluated these aspects in CpG/c activated CLL cells. Stimulated CLL cells increased expression of cIg compared to control treated cells as detected by intracellular immunofluorescence staining for cIg (**Fig. 2A**). Further, using ELISA we demonstrated that CpG/c stimulation caused an increase in secreted IgM in the majority of CLL patients, while IgG levels remained low in both stimulated and control supernatants. CpG/c stimulated samples secreted an average of 1265 ng/ml of IgM compared to 156 ng/ml without treatment (n = 14, p = .029, **Fig. 2B**). In order to address the possibility that residual normal B cells were the primary source of the secreted IgM, we next evaluated the κ and λ light chain usage of the secreted Ig. **Figure 2C** importantly demonstrates that CpG/c stimulated CLL cells secreted either κ or λ light chains but not both, which would be expected if normal polyclonal B cells were the source of secreted Ig. In addition, the patterns of light chain secretion precisely matched the known light chain usage of the patients' leukemic B cells in all cases tested. These data support the conclusion that CpG/c stimulation is able to induce CLL B cell differentiation into ISC.

### CLL B cells induced to differentiate into ISC exhibit decreased *LEF1* and increased expression of *PRDM1*

**Figure 3:**
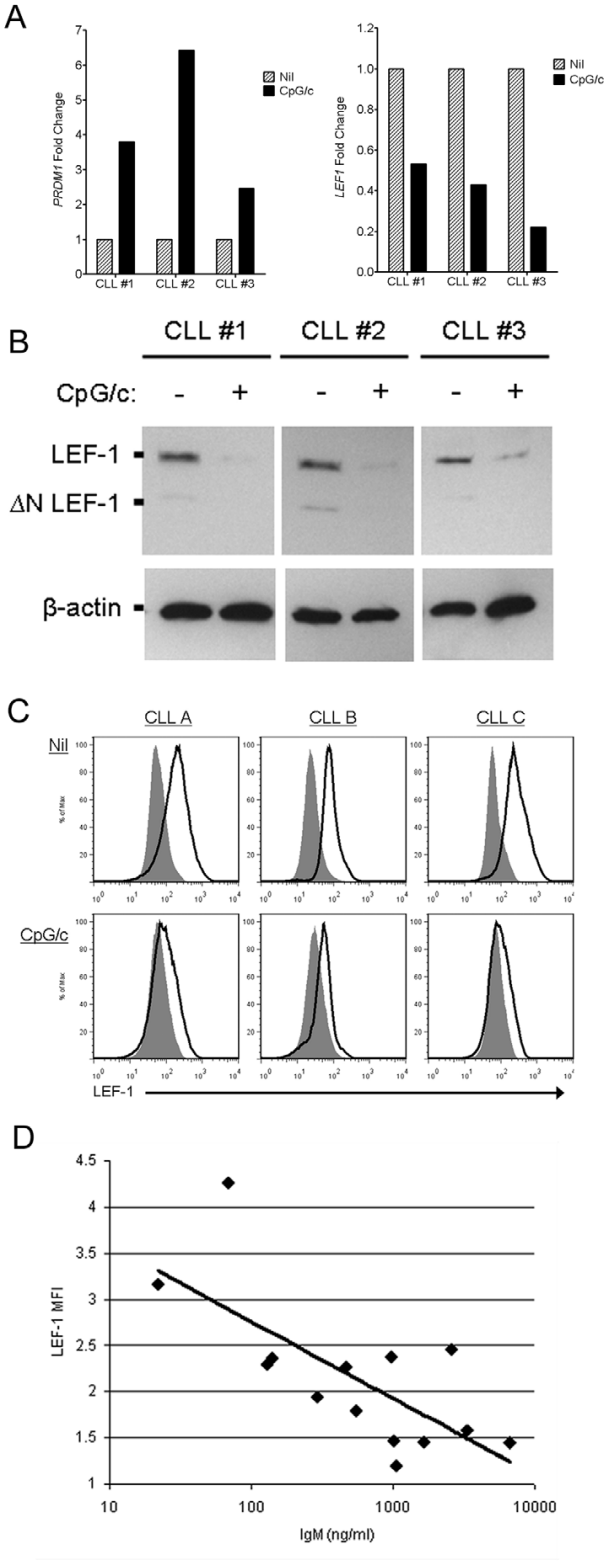
CpG/c stimulated CLL B cells decrease LEF-1 expression and increase PRDM1 expression. **A.** Detection of *PRDM1* (left) and *LEF1* (right) mRNA levels by qPCR in 3 CLL patients in nil or CpG/c treated CLL cultures after 5 days. Transcript levels were normalized to 18 s rRNA and relative fold change was calculated using the 2∧^−ΔΔCT^ method. **B.** Representative western blot analysis of LEF-1 full length and ΔN LEF-1 isoforms in B cells from 3 CLL samples in nil or CpG/c treated CLL cultures after 5 days of culture; β-actin shown as a loading control of samples. **C.** Representative intracellular FACS analysis for LEF-1 in B cells from 2 CLL samples in nil or CpG/c treated CLL cultures after 5 days; Isotype control histogram is shaded grey; LEF-1 histogram is black. **D.** Inverse correlation between LEF-1 Δ mean fluorescence intensity (ΔMFI) and IgM secretion in 14 CpG/c treated CLL samples (best fit line equation y = −0.3606Ln(x)+4.4207; r^2^ = 0.505).

Plasma cell differentiation has been well characterized and shown to require expression of a number of master regulators, with one of the most well characterized being BLIMP1 (gene name *PRDM1*). Thus, we next evaluated the expression levels of *PRDM1* and *LEF1* in CpG/c stimulated CLL cells that had acquired the ability to secrete Ig. In further support of our conclusion that this stimulation induces CLL B cell differentiation, we observed that CpG/c stimulated cells acquired increased expression of *PRDM1* (**Fig. 3A** **,** left). Importantly, we also observed a concomitant decrease in *LEF1* expression as detected by qPCR (**Fig. 3A** **,** right). Further, decreased LEF-1 expression was also observed at the protein level as revealed by western blot and intracellular FACS analysis (**Figs. 3B and 3C**, respectively). However, we also observed significant patient to patient variability in the decrease of LEF-1 protein expression induced by CpG/c. Interestingly, the level of Ig secretion by differentiated CLL cells was observed to inversely correlate with the level of LEF-1 expression remaining in differentiated CLL cells (n = 14, R^2^ = 0.505, **Fig. 3D**). These data support the conclusion that differentiation of CLL B cells into ISC leads to the downregulation of LEF-1 expression.

### Differentiated CLL B cells exhibit decreased activation of the Wnt pathway and decreased survival

**Figure 4:**
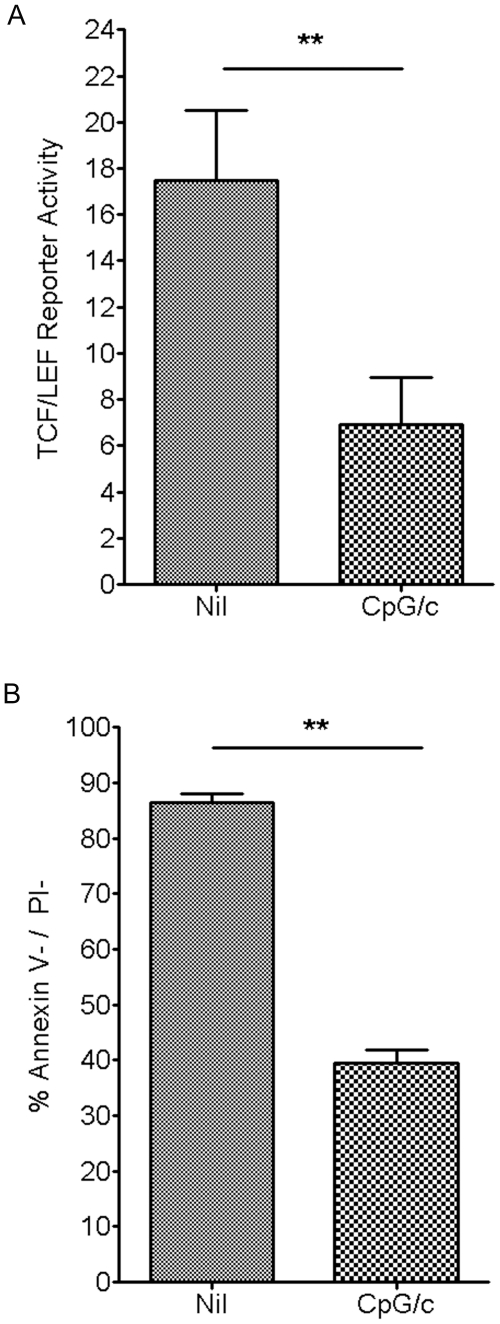
CpG/c stimulated CLL B cells exhibit decreased survival and decreased activation of the Wnt pathway. **A.** TCF/LEF dual luciferase reporter assay relative fold change was measured in samples from 3 CLL patients in nil or CpG/c treated cultures after 5 days of stimulation and then 2 days in media alone (**p = .0098; error bars represent standard error of the mean). **B.** CLL percent survival 48 hours post-ficoll isolation of 5 day CpG/c stimulated or untreated samples from 3 CLL patients (*p = .0039; error bars represent the standard error of the mean).

Previously, we have shown that CLL B cells exhibit aberrant activation of the canonical Wnt signaling pathway as detected using the TCF/LEF dual luciferase reporter assay, while healthy donor B cells lack activation of this pathway.[5] Because LEF-1 is a central mediator of the Wnt pathway and because differentiation of CLL B cells into ISC resulted in decreased expression of LEF-1, we next evaluated the level of Wnt pathway activation in these cells. Indeed, Ig secreting differentiated CLL cells were observed to have decreased activation of the TCF/LEF dual luciferase reporter assay compared to untreated CLL cells. The average fold change in reporter activity was 6.9 for ISC differentiated CLL vs 17.5 for untreated CLL cells (n = 3, p = 0.0098, **Fig. 4A**). This is a 60% decrease in the level of Wnt pathway activation following CLL B cell differentiation. These data support the conclusion that differentiation of CLL cells leads to a robust decrease in Wnt-mediated pro-survival signaling.

LEF-1 and the Wnt pathway are known to regulate survival in numerous cell types.[29], [30] We and others have previously shown that expression of this transcription factor by CLL cells is required for cellular survival.[5], [6] Therefore, we next wanted to evaluate whether ISC differentiated CLL cells that have decreased LEF-1 expression, also display decreased *in vitro* survival. To overcome possible early effects of CpG/c stimulation on apoptosis, we differentiated the cells for 5 days before separating live cells by Ficoll gradient centrifugation and replating equal numbers of differentiated or control cells in AIM-V media for an additional 48 hour incubation period. At this point, cell survival was analyzed and our data demonstrate that ISC differentiated CLL B cells exhibited poorer survival than did untreated CLL cells. Untreated CLL cells had an average of 86.4% surviving cells compared to an average of 39.4% surviving cells for differentiated cells (n = 3, p = 0.0039, **Fig. 4B**). Thus, there was a 55% average decrease in survival following CLL B cell differentiation into ISC. These data support the conclusion that differentiation of CLL cells leads to a decrease in cell survival.

### Multiple agents are able to induce CLL B cell differentiation and subsequent decreases in LEF-1 expression

**Figure 5:**
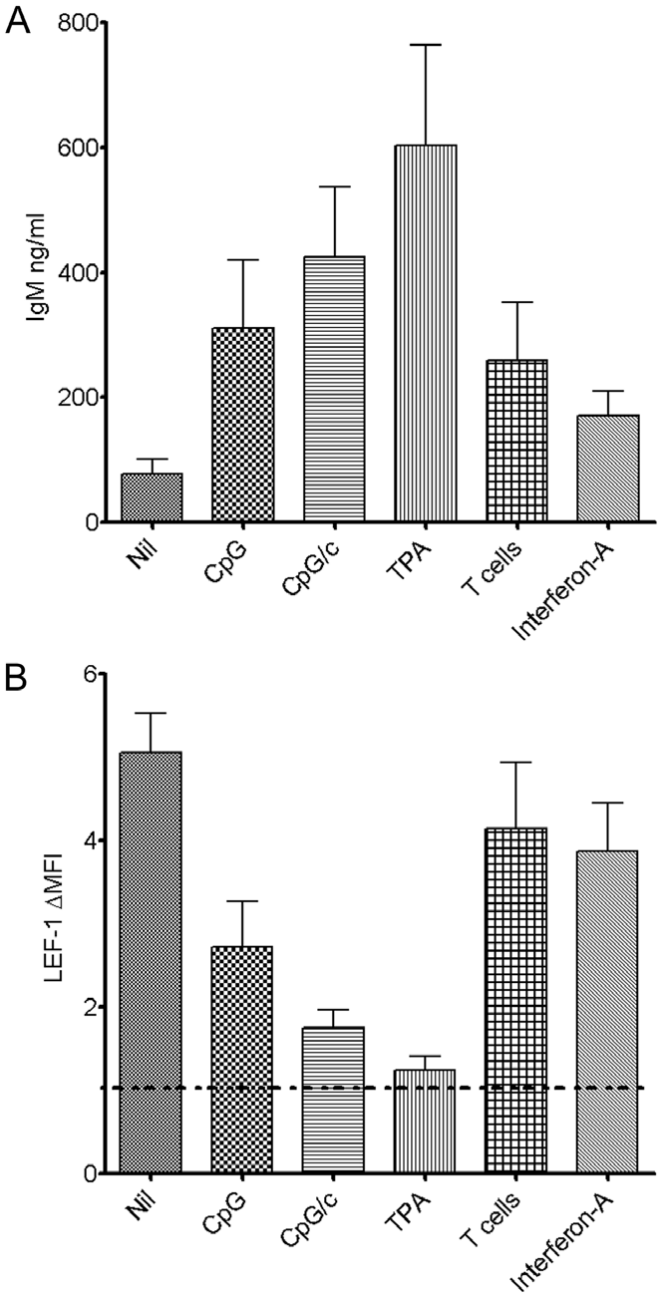
Multiple agents are able to induce CLL B cell differentiation and subsequent decrease in LEF-1 expression. **A.** ELISA detection of IgM levels in cell free culture supernatants from culture of CLL cells from 6 patients. The CLL cells were either unstimulated, or stimulated with CpG, CpG/c, TPA, interferon-alpha, or activated T cells for 5 days of culture (error bars represent standard error of the mean). **B.** Intracellular FACS LEF-1 Δ mean fluorescence intensity (ΔMFI) was measured in the same samples (error bars represent standard error of the mean, dashed line represents no increase in anti-LEF-1 staining over isotype control).

Previously, others had reported the ability of TPA and T cell dependent pokeweed mitogen to induce CLL cell differentiation into ISC.[19], [20] Because CpG/c has the ability to differentiate CLL cells into ISC and downregulate LEF-1, we questioned whether other agents might also possess this ability. To address this point, we tested the ability of CpG alone, CpG/c, TPA, interferon-α, and activated T cells to stimulate CLL cell Ig secretion. TPA stimulation induced the largest increase in Ig secretion, but all stimulations led to a variable increase in Ig secretion (n = 6, **Fig. 5A**). We next evaluated the ability of these stimulations to induce down regulation of LEF-1. Of note, TPA was able to induce the largest decrease in LEF-1 levels, paralleling the robust Ig secretion stimulated by this agent. Moreover, each of the other stimuli also led to decreases in LEF-1 levels, with the degree of LEF-1 expression being associated with the overall levels of secreted Ig (n = 6, **Fig. 5B**). These data support the conclusion that a variety of biological agents capable of stimulating CLL cell differentiation lead to a decrease in LEF-1 expression.

## Discussion

Previously, we identified a pathogenic role for LEF-1 and the Wnt pathway in CLL B cells. In this study, we demonstrate that the differentiation of CLL cells into ISC leads to a decrease in LEF-1 expression and a decrease in TCF/LEF reporter activation. Although other investigators have shown that CLL cells can be induced to differentiate into ISC using a variety of stimuli,[18], [19], [20], [21], [22] we show for the first time that differentiated CLL cells exhibit diminished cell survival. Our study supports the rationale to identify therapeutic agents that are able to induce the differentiation of CLL cells. These findings are timely due to the known challenge of identifying therapeutic strategies to treat refractory CLL.[31]

Our study is the first to uncover the utility of toll-like receptor agonists in the induction of CLL ISC differentiation. We provide evidence that CpG and CpG/c stimulation induce CLL B cells to take on an ISC-like morphology, increase cytoplasmic Ig expression, and secrete light chain restricted Ig. There was patient to patient variability in the response elicited by CpG or CpG/c, with a trend toward patients with worse prognosis (i.e., unmutated Ig variable region) having a more robust response to TLR9 stimulation (data not shown). If this heightened TLR responsiveness remains *in vivo* for CLL patients, then CpG therapy with or without other conventional drugs could be more effective in poor prognosis patients. These findings add a new dimension to our understanding of the effects of TLR agonists on CLL cells and further support the use of TLR agonists in this mature B cell malignancy.

LEF-1 is expressed by B cell precursors in the bone marrow and is critical during B cell development.[8] Mature B and plasma cells lose expression of LEF-1 during B cell development.[9] Similarly, this study has demonstrated that stimulating CLL cells to acquire a further stage of differentiation, i.e., ISC, leads to a decrease in LEF-1 expression while the plasma cell specific transcription factor, PRDM1, increases. We further verified the decrease in LEF-1 protein via western blot and intracellular FACS analysis. Interestingly, there was an inverse correlation between the level of LEF-1 expression and the amount of Ig secreted by the differentiated leukemic B cells. These observations suggest that agents with the greatest ability to induce CLL differentiation (i.e., Ig secretion) would also be most effective in decreasing LEF-1 expression and CLL survival.

The canonical Wnt signaling pathway culminates in the nucleus with alterations in transcriptional activity. As LEF-1 is a mediator of this activity and is also decreased by the differentiation of CLL cells, we hypothesized that there would be a decrease in the level of Wnt pathway activation following CLL differentiation. Indeed, TCF/LEF dual luciferase reporter assay activity was decreased following CLL cell differentiation into ISC. Further, we have shown that differentiated CLL cells exhibit decreased survival compared to untreated, non-differentiated cells. As CpG has also been shown to directly induce apoptosis in CLL cells,[15] these direct and differentiation-induced effects of CpG on cell survival could synergize for an effective therapy for this mature B cell malignancy. Although the precise mechanisms that underlie differentiation induced diminished survival requires further study, it is intriguing to note that there may be a role for autophagy in immune cell differentiation and cell death.[32]

Indeed, differentiation therapy has been successfully used in certain hematological malignancies[10] and is being investigated in the setting of cancer stem cells as a means to eradicate this tumor population.[33], [34] Differentiation therapy has many attractive properties which include a potentially more gentle effect on non-tumor cells of the patient compared to chemotherapy and sensitization of differentiated cells to more conventional therapy. The use of TLR agonists in hematopoietic malignancies has the potential to aid any existing or augmented anti-leukemic immune response and to sensitize the leukemic cells to such a response. In addition, we speculate that TLR agonists could sensitize CLL B cells to conventional drugs. In summary, our data suggest that the role for TLR agonists as therapeutic agents in CLL needs to be further investigated along with studies into additional differentiation agents that could lead to possible therapies in CLL.
